# Pericardial Parietal Mesothelial Cells: Source of the Angiotensin-Converting-Enzyme of the Bovine Pericardial Fluid

**DOI:** 10.5935/abc.20170155

**Published:** 2017-11

**Authors:** Ilsione Ribeiro de Sousa Filho, Isabela Cabral Cavicchioli Pereira, Lourimar José de Morais, Livia das Graças Vieito Lombardi Teodoro, Maria Laura Pinto Rodrigues, Roseli Aparecida da Silva Gomes

**Affiliations:** Universidade Federal do Triângulo Mineiro (UFTM), Uberaba MG - Brazil

**Keywords:** Renin-Angiotensin System, Peptidyl-Dipeptidase A, Pericardial Fluid, Hypertrophy Left Ventricular, Cattle

## Abstract

**Background:**

Angiotensin II (Ang II), the primary effector hormone of the renin-angiotensin system (RAS), acts systemically or locally, being produced by the action of angiotensin-converting-enzyme (ACE) on angiotensin I. Although several tissue RASs, such as cardiac RAS, have been described, little is known about the presence of an RAS in the pericardial fluid and its possible sources. Locally produced Ang II has paracrine and autocrine effects, inducing left ventricular hypertrophy, fibrosis, heart failure and cardiac dysfunction. Because of the difficulties inherent in human pericardial fluid collection, appropriate experimental models are useful to obtain data regarding the characteristics of the pericardial fluid and surrounding tissues.

**Objectives:**

To evidence the presence of constituents of the Ang II production paths in bovine pericardial fluid and parietal pericardium.

**Methods:**

Albumin-free crude extracts of bovine pericardial fluid, immunoprecipitated with anti-ACE antibody, were submitted to electrophoresis (SDS-PAGE) and gels stained with coomassie blue. Duplicates of gels were probed with anti-ACE antibody. In the pericardial membranes, ACE was detected by use of immunofluorescence.

**Results:**

Immunodetection on nitrocellulose membranes showed a 146-KDa ACE isoform in the bovine pericardial fluid. On the pericardial membrane sections, ACE was immunolocalized in the mesothelial layer.

**Conclusions:**

The ACE isoform in the bovine pericardial fluid and parietal pericardium should account at least partially for the production of Ang II in the pericardial space, and should be considered when assessing the cardiac RAS.

## Introduction

Cardiovascular diseases are the major cause of morbidity and mortality worldwide.^1^ It has been well established that dysregulation or overexpression of the renin-angiotensin system (RAS) leads to several harmful vascular effects, contributing to the pathophysiology of cardiovascular diseases.^2^ Angiotensin II (Ang II) is the primary effector hormone of that system, produced by the action of angiotensin-converting-enzyme (ACE) on its substrate, angiotensin I (Ang I).^3-5^ Angiotensin II can act systemically or as a tissue factor, locally produced. Tissue Ang II has paracrine and autocrine actions, promoting cell growth, apoptosis, inflammation, oxidative stress and tissue damage, leading to hypertrophy, fibrosis, heart failure and cardiac dysfunction.^6,7^ Local tissue^6,8^ and intracellular^9^ RASs, such as cardiac RAS, have been described, although little is known about the presence of an RAS in the pericardial fluid and its possible sources. Angiotensin II, some growth factors and enzymes have been identified in that fluid.^10^ Gomes et al.^11^ have shown ACE activity in the human pericardial fluid, and Bechtloff et al.^12^ have shown the presence of the protein fraction of ACE in the pericardial fluid of patients with coronary artery disease. However, the source of that enzyme in the pericardial fluid remains unknown.

Because of the difficulties inherent in pericardial fluid collection, the use of appropriate experimental models is essential. The heart is contained inside a fibroserous sac, the pericardial sac, which has an inner layer, the serous pericardium, which delimits the pericardial cavity. Serous pericardium has a visceral membrane, inseparable from the heart, and a parietal membrane, continuous with the visceral one. The pericardial fluid is found inside that cavity.^13,14^

Therefore, characterization in animal models of the pericardial fluid and surrounding tissues, including the source of the macromolecules of that fluid, is essential so that the results can be translated to human beings. This study aimed at collecting evidence in the bovine pericardial parietal membrane and pericardial fluid of the presence of constituents of the Ang II production paths.

## Methods

### Collection of bovine pericardial fluid and parietal pericardium

This study used fragments of pericardial parietal membranes, as well as pericardial fluid, of six Nelore cattle (*Bos indicus*, 1758) collected in Delta slaughterhouse (Delta-MG), subject to authorization by the veterinarians in charge. The fragments of pericardial membranes collected were washed and conditioned in saline solution at 4ºC. The pericardial fluids, aspirated from the pericardial cavities with 20-mL sterile syringes, were maintained at 4ºC and, with the membranes, transported to the laboratory.

Because this study was performed "ex vivo", it required no submission to the Ethics Committee in the Use of Animals (CEUA) UFTM, according to the Inner Regulation of the CEUA/UFTM, article 2, subsection I, §2º.

### Processing of pericardial parietal membranes

The fragments of the pericardial parietal membranes were washed in saline solution and dissected in horizontal laminar flow (Labconco, USA), in nutrient DMEM medium, to remove the adipose tissue of the epipericardial layer of the parietal pericardium. Then they were washed in TBS and sliced into fragments of approximately 1.0x0.3 cm, which were embedded in a cryoprotective medium (OCT) and submitted to frozen fixation with liquid nitrogen. After fixation, the fragments were sectioned in a cryostat (Leica Microsystems), and the 2-µm sections obtained were mounted in glass slides, fixed in acetone for 10 minutes, and stored at -20ºC.

### Pericardial fluid processing

The pericardial fluid was transferred to microcentrifuge tubes and centrifuged at 14000 rpm and 4ºC for 10 minutes (Centrifuge 5402, Eppendorf). The supernatants were collected, and the clear ones, with no visual blood contamination, were used, constituting a pericardial fluid pool.

The high concentrations of albumin in the pericardial fluid were reduced by using blue agarose resin (Bio-Rad). Samples of 250 µL of crude extract of pericardial fluid were incubated with 1 mL of blue agarose, balanced with sodium phosphate buffer 0.05 M, pH 6. They were incubated for 2 hours, at room temperature, under agitation. Then, the pericardial fluid extracts were centrifuged at 14000 rpm and 4ºC, and the supernatants were collected for further use.

### Immunoprecipitation of pericardial fluid

Samples of 500 µL of pericardial fluid, obtained after removing albumin, were incubated with 2.5 µL of anti-ACE antibody (200 µg/mL, Santa Cruz), during the night. Then, 25 µL of CL-4B Sepharose spheres (Amersham Biosciences) conjugated with G protein were added to the samples and incubated for 2 hours. The suspensions were centrifuged for 5 minutes at 14000 rpm. All procedures were performed under agitation at 4ºC. The supernatants were discarded and the precipitates collected were diluted with 20 µL of the sample solution.^15^ The immunocomplexes obtained were analyzed with SDS polyacrylamide gel electrophoresis (SDS-PAGE).

### SDS polyacrylamide gel electrophoresis (SDS-PAGE)

The immunocomplexes obtained were diluted with the sample solution under reducing conditions, at 70ºC, centrifuged and applied to gels at 7.5% concentration. The polypeptide bands were separated by use of SDS-PAGE (Mighty Small II SE 260, Amersham Biosciences) at constant 25-mA current. The gels obtained were fixed, stained with coomassie blue and bleached. Duplicates of non-fixed gels were transferred to the nitrocellulose membrane (Invitrogen), in TE 22 (Amersham Biosciences) transfer unit, containing modified Towbin buffer,^16^ under agitation, during the night, at 4ºC, with a constant 200-mA current. The membranes obtained were stained with Ponceau S to assess the presence of polypeptide fractions, bleached with distilled water, dried in filter paper and submitted to ACE immunodetection.

### Immunodetection in nitrocellulose membranes

The nitrocellulose membranes were incubated with 10% skim milk and 2% serum bovine albumin in Tris-buffered saline (TBS), during the night, under agitation, at 4ºC, to block nonspecific bindings. Then, that solution was replaced with another containing the primary anti-ACE antibody (200 µg/mL, Santa Cruz), diluted at 1:100, and the membranes were incubated for 2 hours. After incubation with the primary antibody, the membranes were extensively washed with TBS and incubated with the secondary antibody [F(ab')_2_], rabbit anti-IgG, conjugated with peroxidase (Amersham), diluted at 1:1000, for 2 hours. The membranes were washed again, and the immunoreactive bands were revealed in a solution containing diaminobenzidine (DAB, Dako). The revelation was inactivated in distilled water. All antibodies were diluted in a solution of 1% bovine serum albumin and 0.05% Tween 20 in TBS, the incubation with antibodies being performed at room temperature under agitation. To determine the specificity of the reaction, the membranes were incubated without the primary antibody.

### Immunofluorescence in pericardial parietal membranes

The pericardial parietal sections obtained with the cryomicrotome were washed in TBS and incubated with anti-ACE antibody (200 µg/mL, Santa Cruz), for 1 hour, at room temperature, in a dark humid chamber. After incubation with primary antibody, the sections were washed in TBS plus 0.05% Tween 20 several times and incubated with rabbit anti-IgG secondary antibody conjugated with rhodamine (Alexa Fluor Molecular Probes 568). After being extensively washed, the sections mounted with Fluoromount G (Southern Biotech) were observed and documented under a fluorescence microscope Olympus, with a 568-nm wave length. To determine the immunostaining specificity, control sections were incubated without the primary antibody.

## Results

### ACE detection in the bovine pericardial fluid

**Figure 1:**
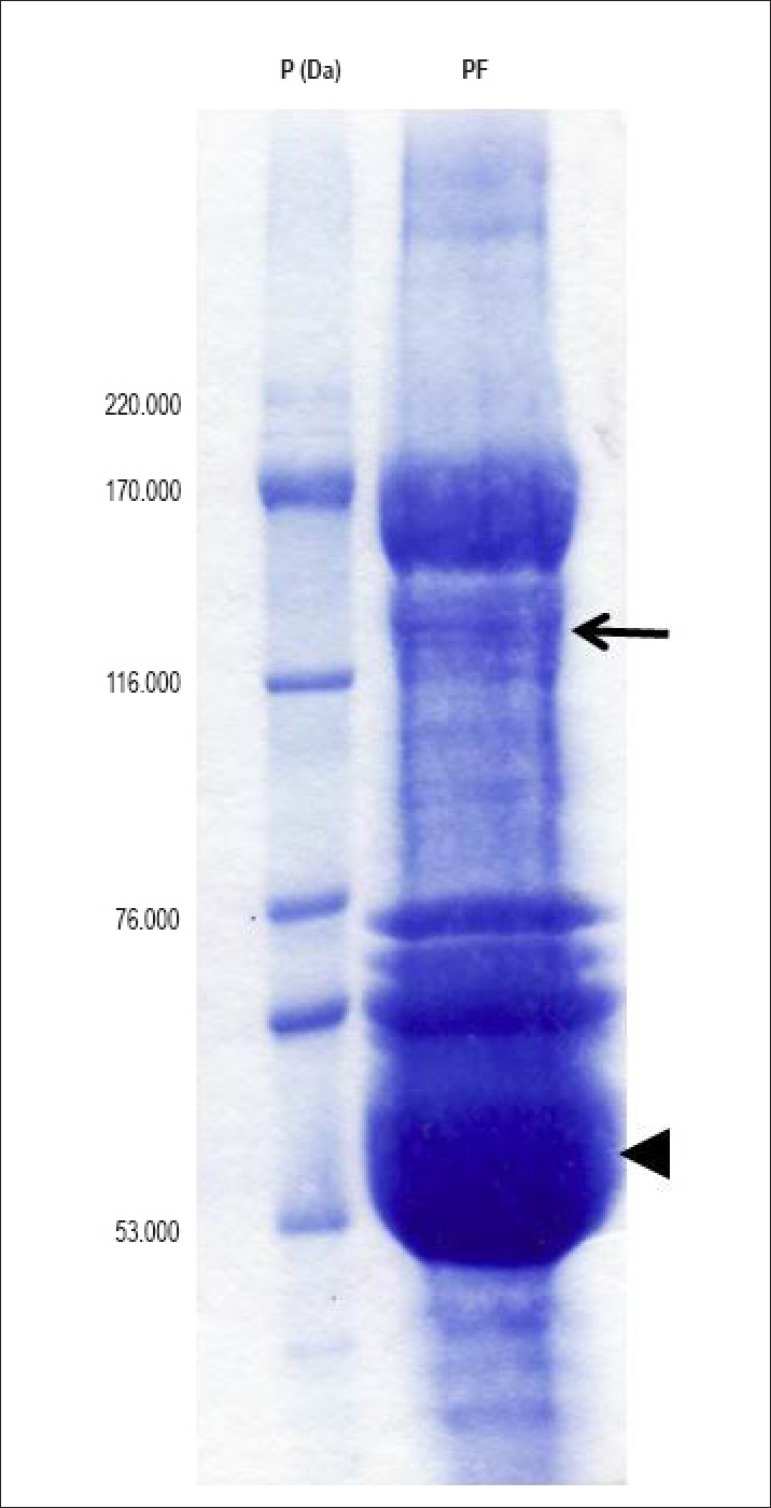
SDS-PAGE of samples of bovine pericardial fluid immunoprecipitated with anti-ACE antibody. Representative gel (7.5%), stained with coomassie blue, showing the presence of a band with apparent molecular mass of 146 kDa (arrow), suggestive of an ACE isoform. The arrow head indicates IgG heavy chain. These results are representative of three independent experiments. PF: pericardial fluid; P (Da): patterns of molecular weights.

**Figure 2:**
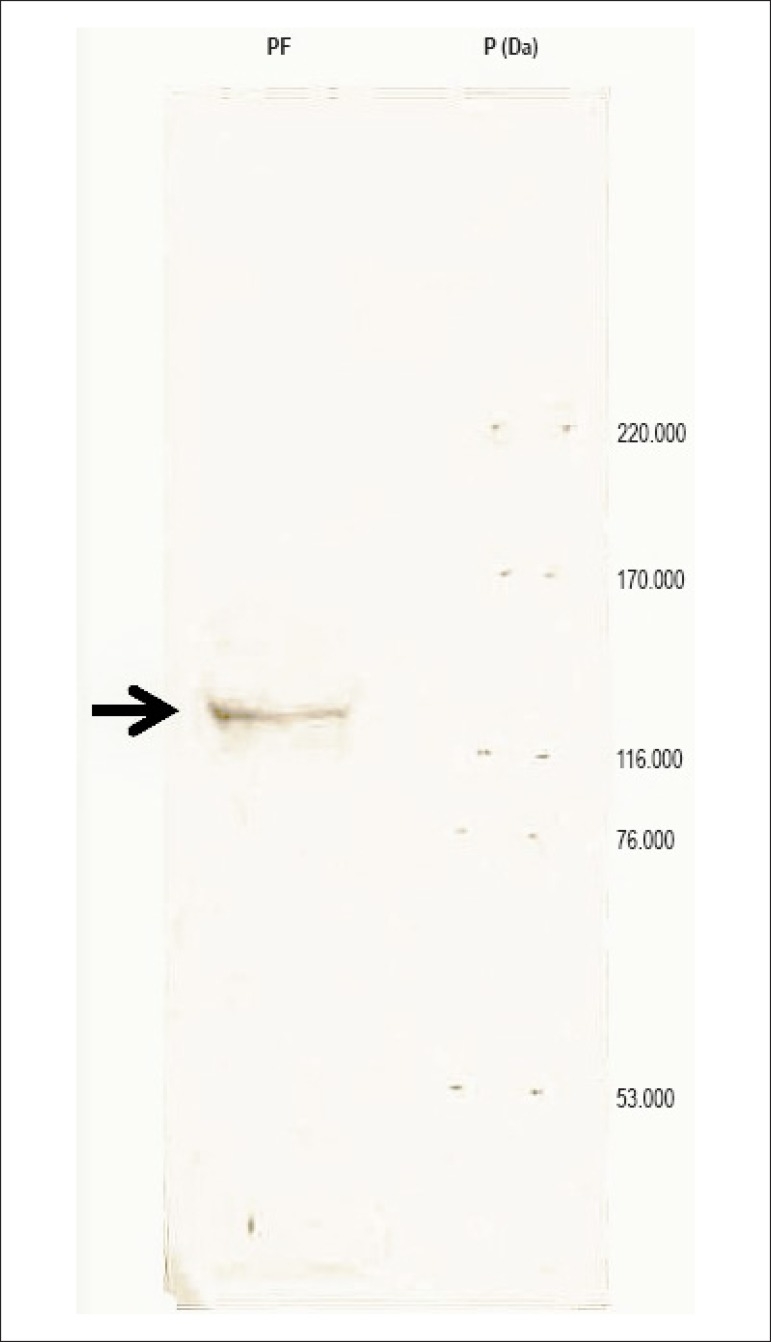
Western Blot of samples of bovine pericardial fluid immunoprecipitated with anti-ACE antibody. The polypeptide fractions separated by SDS-PAGE were transferred to the nitrocellulose membranes and probed with anti-ACE antibody. The head indicates the immunomarked ACE isoform. These results are representative of three independent experiments. PF: pericardial fluid; P (Da): patterns of molecular weights.

When the crude extracts of the pericardial fluid underwent immunoprecipitation with anti-ACE antibody and were analyzed with SDS-PAGE under reducing conditions, a band with molecular mass of approximately 146 kDa, similar to the ACE mass (Figure 1; arrow), was detected. Of the polypeptide bands observed, the most prominent was the IgG heavy chain, because the antibody was not removed after being added to the pericardial fluid during immunoprecipitation (Figure 1; arrow head). In addition, other thinner bands were noted and could have been co-immunoprecipitated or even not properly removed by washing with buffer. The bovine pericardial fluid ACE immunoprecipitation was confirmed with immunodetection in the nitrocellulose membranes, which evidenced the ACE isoform in the position expected for an enzyme (Figure 2; arrow).

### Immunolocalization of ACE in the pericardial membrane

**Figure 3:**
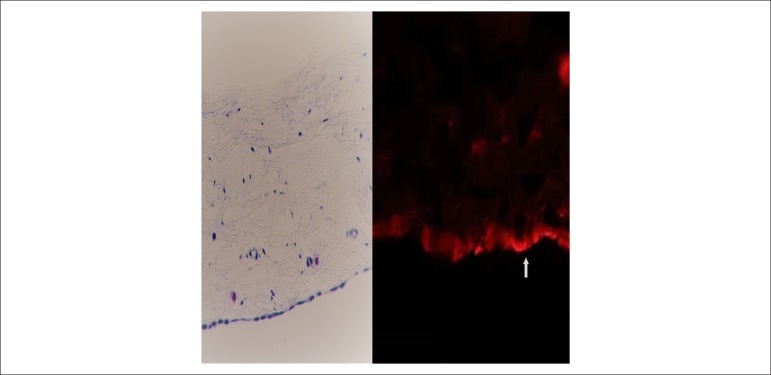
Right: image representative of sections of bovine pericardial parietal membranes, cryofixed and submitted to ACE immunodetection. Note the positive mesothelial layer (arrow). Left: histological section of bovine parietal pericardium stained with toluidine blue. Original magnification: 40x.

The histological sections of the parietal pericardium submitted to ACE detection by use of immunofluorescence showed unequivocal positivity for ACE in the mesothelial cells (Figure 3, right). That positivity neither was continuous in the entire mesothelium nor had the same intensity. Specific fluorescence for ACE was not observed in the fibrous layer of the pericardial membrane, except for the blood vessels, because ACE is expressed in endothelial cells. Negative controls showed no staining. Figure 3 shows the histological section of the parietal pericardium stained with toluidine blue.

## Discussion

The present study evidences the presence of an ACE isoform in bovine pericardial fluid, and establishes the ACE location in mesothelial cells of the bovine pericardial parietal membrane, for the first time, indicating that membrane as a possible source of the pericardial fluid ACE.

The RAS, originally characterized as a circulating endocrine system, comprises several enzymatic paths and bioactive components that have several functions.

Currently, there is plenty of evidence of the presence of tissue RASs that influence local cell actions, with intracellular and subcellular components.^17-20^ Local RASs have been shown in many tissues/organs, such as heart, kidneys, adrenal glands, blood vessels, pancreas, liver, brain, and adipose tissue.^6,19,21-24^ Regarding the cardiac RAS, several of its constituents, such as angiotensinogen, renin, ACE, Ang I and Ang II, and AT1 and AT2 receptors, were detected in different regions of the heart, such as the atria, conduction system, heart valves, coronary arteries and ventricles, being synthesized by different cell types, such as fibroblasts and myocytes.^6,24-26^

The importance of the pericardium and pericardial fluid to control cardiac function has been established in past years. The quiescent nature of the visceral and parietal pericardium has been questioned, resulting in evidence for an important role in the production of substances that could have paracrine actions on the heart. When the human parietal pericardium is compared with that of other species, we observe that bovine parietal pericardium has the closest histological constitution to that of the human species.^27,28^ Thus, characterizing the bovine pericardium, mainly the macromolecules and mediators produced by the cells that delimit the pericardial cavity, is paramount to the better understanding of the biology and importance of that membrane and of the pericardial fluid under physiological conditions or in association with any disease.

The pericardial fluid is considered an ultrafiltrate of plasma, added by some components of the myocardial interstitial fluid. Its protein concentration is lower than that of the plasma, but with a relatively high albumin concentration.^14^ Substances detected in the human or animal pericardial fluid, such as endothelin-1, beta fibroblast growth factor (bFGF), Ang II, renin, atrial natriuretic factor, vascular endothelial growth factor (VEGF), interleukin-6, and cell adhesion molecules, could act upon the heart.^10,29-31^ Modulation of growth and survival of cardiac myocytes,^10^ endothelial cells and smooth muscle cells^32,33^ are some biological effects of mediators existing in the pericardial fluid of patients with ischemic and non-ischemic cardiac diseases. Limana et al.^34^ have shown that, in response to myocardial infarction, epicardial c-kit+ cells reactivate an embryonic program, in which soluble factors of the pericardial fluid play a fundamental role. Thus, the knowledge about that fluid composition has pathophysiological importance and diagnostic significance.^30^

Our results evidence the presence of an ACE isoform in the bovine pericardial fluid, showing the existence of a part of the RAS in the pericardial cavity, probably of local origin. Although the pericardial fluid is a plasma ultrafiltrate and plasma mediators, such as Ang II, can spread to the pericardial fluid with no restriction, the same does not happen with ACE. The structural organization of the mesotelial layer of the pericardium, both parietal and visceral ones, prevent that free circulation. The presence of tight junctions between the mesothelial cells^27,28,35^ prevents paracellular transport of macromolecules with molecular mass above 40 KDa.^36^ Because the ACE isoform present in the bovine pericardial fluid has molecular mass of approximately 146 KDa, similar to that predicted for human ACE,^5^ the paracellular route would not be an access way to the pericardial cavity.

In addition to the above cited factors, which partially support the local synthesis of ACE, ACE localization should be considered. Immunofluorescence evidenced positivity in parietal pericardial mesothelial cells and in the blood vessels of the pericardial membrane. Immunolocalization of ACE in blood vessels was expected, because ACE has ubiquitous distribution in the endothelium.^5,37^ However, in mesothelial cells, its immunolocalization is a strong evidence that those cells are the source of pericardial fluid ACE, because: *i*) they have a close anatomical relationship with the pericardial cavity, because they delimit it; *ii*) ACE is an integral protein of the membrane, being, thus, produced by mesothelial cells, with its extracellular domain directed to the pericardial cavity;^5^ *iii*) the ability of mesothelial cells to synthesize ACE has been demonstrated by the presence of that enzyme's mRNA in cultured human peritoneal mesothelial cells, by use of RT-PCR;^38^ *iv*) they have abundant endoplasmic reticulum and developed Golgi complex,^27,28,35^ consistent with the profile of cells capable of active protein synthesis.

Corroborating with those arguments, several studies have shown the metabolic profile of mesothelial cells. Mesothelial cells synthesize and secrete lubricants, such as glycosaminoglycans and surfactant, to prevent friction and the formation of adhesions between the parietal and visceral surfaces.^39^ They play a critical role in homeostasis control of the serous membranes in response to injury, inflammation and immunoregulation.^39^ In addition, mesothelial cells play a central role in the repair of serous membranes, secretion of inflammatory mediators, chemokines, growth factors and extracellular matrix components. They have different phenotypes, which, depending on their location and activation status, reflect functional differences.^39^

The importance of local RASs has not been totally clarified. Higher concentrations of active cardiovascular mediators in the pericardial fluid than in the plasma raises a question about their origin and possible actions upon the surrounding tissues. The pericardial fluid of patients with coronary artery disease trigger substantial arterial contractions in isolated carotid arteries of rats, which are mediated primarily by ET-1.^40^ Our results showed both the presence of an ACE isoform in the pericardial fluid, and, for the first time, the immunolocalization of that protein in parietal pericardial mesothelial cells, suggesting that the parietal pericardial mesothelial layer is one possible source of the pericardial fluid ACE. Thus, the Ang II produced locally could act on its own pericardial mesothelial cells, both parietal and visceral, or even directly on the myocardium, promoting inflammation, oxidative stress and cell death, contributing to cardiac hypertrophy and fibrosis. In addition, it could act directly on the myocardial microcirculation promoting important vasomotor effects. In that context, the pericardial fluid would be an important reservoir of mediators that could modulate the functions of cardiac cells.

The use of experimental models with tissues similar to the human ones, both regarding structural organization and cell constitution, would be more suitable for studying certain human conditions. In addition to structural organization, biochemical and molecular features are fundamental to achieving optimal balance between quantity and quality of the data produced and their relevance for the condition investigated.

The structural features of the bovine pericardial mesotelial layer, similar to the human ones,^27,28^ suggest our results can be extended to human pericardial mesothelial cells, which could be the partial source of the human pericardial fluid ACE.^11,12^ A better knowledge of both the pericardial fluid constituents and the mesothelial cells in proper animal models could help understanding the paracrine or autocrine effects of mediators produced by the pericardium on the heart.

One limitation of our study was the volume of the pericardial fluid obtained from the animals in the sample. Because of the difficulties inherent in collecting bovine pericardial fluid, the volume was relatively low. Thus, further research is required to clarify how mesothelial cells interact with their local environment and what is their contribution to the production of mediators in the pericardial fluid that can modulate cell actions essential to maintain cardiac homeostasis.

## Conclusions

The Ang II present in the bovine pericardial fluid is partially produced by the action of the ACE existing in that fluid, pericardial parietal mesothelial cells being a source of that ACE.

## References

[1] World Health Organization (2011). Global atlas on cardiovascular diseases prevention and control.

[2] Dzau VJ (2001). Theodore Cooper Lecture - Tissue angiotensin and pathobiology of
vascular disease: a unifying hypothesis. Hypertension.

[3] Acharya KR, Sturrock ED, Riordan JF, Ehlers MR (2003). Ace revisited: a new target for structure-based drug
design. Nat Rev Drug Discov.

[4] Riordan JF (2003). Angiotensin-I-converting enzyme and its relatives. Genome Biol.

[5] Bernstein KE, Ong FS, Blackwell WL, Shah KH, Giani JF, Gonzalez-Villalobos RA (2012). A modern understanding of the traditional and nontraditional
biological functions of angiotensin-converting enzyme. Pharmacol Rev.

[6] Paul M, Poyan Mehr A, Kreutz R (2006). Physiology of local renin-angiotensin systems. Physiol Rev.

[7] Sun Y (2009). Myocardial repair/remodelling following infarction: roles of
local factors. Cardiovasc Res.

[8] Re RN (2004). Mechanisms of disease: local renin-angiotensin-aldosterone
systems and the pathogenesis and treatment of cardiovascular
disease. Nat Clin Pract Cardiovasc Med.

[9] Kumar R, Singh VP, Baker KM (2007). The intracellular renin-angiotensin system: a new
paradigm. Trends Endocrinol Metab.

[10] Corda S, Mebazaa A, Gandolfini MP, Fitting C, Marotte F, Peynet J (1997). Trophic effect of human pericardial fluid on adult cardiac
myocytes. Differential role of fibroblast growth factor-2 and factors
related to ventricular hypertrophy. Circ Res.

[11] Gomes RA, Ld Teodoro, Lopes IC, Bersanetti PA, Carmona AK, Hial V (2008). Angiotensin-converting enzyme in pericardial fluid: comparative
study with serum activity. Arq Bras Cardiol.

[12] Bechtloff R, Goette A, Bukowska A, Kähne T, Peters B, Huth C (2011). Gender and age-dependent differences in the
bradykinin-degradation within the pericardial fluid of patients with
coronary artery disease. Int J Cardiol.

[13] Michailova KN, Usunoff KG (2006). Serosal membranes (Pleura, Pericardium, Peritoneum): normal
structure, development and experimental pathology. Adv Anat Embryol Cell Biol.

[14] Holt JP (1970). The normal pericardium. Am J Cardiol.

[15] Laemmli UK (1970). Cleavage of structural proteins during the assembly of the head
of bacteriophage T4. Nature.

[16] Towbin H, Gordon J (1984). Immunoblotting and dot immunobinding--current status and
outlook. J Immunol Methods.

[17] Abadir PM, Walston JD, Carey RM (2012). Subcellular characteristics of functional intracellular
renin-angiotensin systems. Peptides.

[18] Carey RM (2012). Functional intracellular renin-angiotensin systems: potential for
pathophysiology of disease. Am J Physiol Regul Integr Comp Physiol.

[19] Kobori H, Nangaku M, Navar LG, Nishiyama A (2007). The intrarenal renin-angiotensin system: from physiology to the
pathobiology of hypertension and kidney disease. Pharmacol Rev.

[20] Kumar R, Thomas CM, Yong QC, Chen W, Baker KM (2012). The intracrine renin-angiotensin system. Clin Sci.

[21] Baltatu OC, Campos LA, Bader M (2011). Local renin-angiotensin system and the brain--a continuous quest
for knowledge. Peptides.

[22] Carey RM, Siragy HM (2003). Newly recognized components of the renin-angiotensin system:
potential roles in cardiovascular and renal regulation. Endocr Rev.

[23] Cheng Q, Leung PS (2011). An update on the islet renin-angiotensin system. Peptides.

[24] Dostal DE, Baker KM (1999). The cardiac renin-angiotensin system conceptual or a regulator of
cardiac function. Circ Res.

[25] Dzau VJ, Ellison KE, Brody T, Ingelfinger J, Pratt R (1987). A comparative study of the distribution of renin and
angiotensinogen messenger ribonucleic acids in rat and mouse
tissues. Endocrinology.

[26] Hellmann W, Suzuki F, Ohkubo H, Nakanishi S, Ludwig G, Ganten D (1988). Angiotensinogen gene expression in extrahepatic rat tissues:
application of a solution hybridization assay. Naunyn Schmiedebergs Arch Pharmacol.

[27] Ishihara T, Ferrans VJ, Jones M, Boyce SW, Kawanami O, Roberts WC (1980). Histologic and ultrastructural features of normal human parietal
pericardium. Am J Cardiol.

[28] Ishihara T, Ferrans VJ, Jones M, Boyce SW, Roberts WC (1981). Structure of bovine parietal pericardium and of unimplanted
Ionescu-Shiley pericardial valvular bioprostheses. J Thorac Cardiovasc Surg.

[29] Namiki A, Kubota T, Fukazawa M, Ishikawa M, Moroi M, Aikawa J (2003). Endothelin-1 concentrations in pericardial fluid are more
elevated in patients with ischemic heart disease than in patients with
nonischemic heart disease. Jpn Heart J.

[30] Fujita M, Komeda M, Hasegawa K, Kihara Y, Nohara R, Sasayama S (2001). Pericardial fluid as a new material for clinical heart
research. Int J Cardiol.

[31] Mebazaa A, Wetzel RC, Dodd-o JM, Redmond EM, Shah AM, Maeda K (1998). Potential paracrine role of the pericardium in the regulation of
cardiac function. Cardiovasc Res.

[32] Iwakura A, Fujita M, Hasegawa K, Sawamura T, Nohara R, Sasayama S (2000). Pericardial fluid from patients with unstable angina induces
vascular endothelial cell apoptosis. J Am Coll Cardiol.

[33] Yoneda T, Fujita M, Kihara Y, Hasegawa K, Sawamura T, Tanaka T (2000). Pericardial fluid from patients with ischemic heart disease
accelerates the growth of human vascular smooth muscle cells. Jpn Circ J.

[34] Limana F, Bertolami C, Mangoni A, Di Carlo A, Avitabile D, Mocini D (2010). Myocardial infarction induces embryonic reprogramming of
epicardial c-kit(+) cells: role of the pericardial fluid. J Mol Cell Cardiol.

[35] Mutsaers SE (2002). Mesothelial cells: their structure, function and role in serosal
repair. Respirology.

[36] Page E, Upshaw-Earley J, Goings G (1992). Permeability of rat atrial endocardium, epicardium, and
myocardium to large molecules. Stretch-dependent effects. Circ Res.

[37] Hial V, Gimbrone MA Jr, Peyton MP, Wilcox GM, Pisano JJ (1979). Angiotensin metabolism by cultured human vascular endothelial and
smooth muscle cells. Microvasc Res.

[38] Kyuden Y, Ito T, Masaki T, Yorioka N, Kohno N (2005). Tgf-beta1 induced by high glucose is controlled by
angiotensin-converting enzyme inhibitor and angiotensin II receptor blocker
on cultured human peritoneal mesothelial cells. Perit Dial Int.

[39] Mutsaers SE, Birnie K, Lansley S, Herrick SE, Lim CB, Prêle CM (2015). Mesothelial cells in tissue repair and fibrosis. Front Pharmacol.

[40] Nemeth Z, Cziraki A, Szabados S, Horvath I, Koller A (2015). Pericardial fluid of cardiac patients elicits arterial
constriction: role of endothelin-1. Can J Physiol Pharmacol.

